# Development and validation of a NanoString BASE47 bladder cancer gene classifier

**DOI:** 10.1371/journal.pone.0243935

**Published:** 2020-12-17

**Authors:** Jordan Kardos, Tracy L. Rose, Ujjawal Manocha, Sara E. Wobker, Jeffrey S. Damrauer, Trinity J. Bivalaqua, Max Kates, Kristin J. Moore, Joel S. Parker, William Y. Kim

**Affiliations:** 1 Lineberger Comprehensive Cancer Center, University of North Carolina at Chapel Hill, Chapel Hill, North Carolina, United States of America; 2 Division of Oncology, Department of Medicine, University of North Carolina at Chapel Hill, Chapel Hill, North Carolina, United States of America; 3 Department of Urology, University of North Carolina at Chapel Hill, Chapel Hill, North Carolina, United States of America; 4 Department of Pathology and Laboratory Medicine, University of North Carolina at Chapel Hill, Chapel Hill, North Carolina, United States of America; 5 Department of Urology, James Buchanan Brady Urological Institute, Johns Hopkins University School of Medicine, Baltimore, Maryland, United States of America; 6 Department of Epidemiology, University of North Carolina at Chapel Hill, Chapel Hill, North Carolina, United States of America; National Cancer Institute, UNITED STATES

## Abstract

**Background:**

Recent molecular characterization of urothelial cancer (UC) has suggested potential pathways in which to direct treatment, leading to a host of targeted therapies in development for UC. In parallel, gene expression profiling has demonstrated that high-grade UC is a heterogeneous disease. Prognostic basal-like and luminal-like subtypes have been identified and an accurate transcriptome BASE47 classifier has been developed. However, these phenotypes cannot be broadly investigated due to the lack of a clinically viable diagnostic assay. We sought to develop and evaluate a diagnostic classifier of UC subtype with the goal of accurate classification from clinically available specimens.

**Methods:**

Tumor samples from 52 patients with high-grade UC were profiled for BASE47 genes concurrently by RNAseq as well as NanoString. After design and technical validation of a BASE47 NanoString probeset, results from the RNAseq and NanoString were used to translate diagnostic criteria to the Nanostring platform. Evaluation of repeatability and accuracy was performed to derive a final Nanostring based classifier. Diagnostic classification resulting from the NanoString BASE47 classifier was validated on an independent dataset (n = 30). The training and validation datasets accurately classified 87% and 93% of samples, respectively.

**Results:**

Here we have derived a NanoString-platform BASE47 classifier that accurately predicts basal-like and luminal-like subtypes in high grade urothelial cancer. We have further validated our new NanoString BASE47 classifier on an independent dataset and confirmed high accuracy when compared with our original Transcriptome BASE47 classifier.

**Conclusions:**

The NanoString BASE47 classifier provides a faster turnaround time, a lower cost per sample to process, and maintains the accuracy of the original subtype classifier for better clinical implementation.

## Introduction

Gene expression profiling has demonstrated that high-grade urothelial cancer (UC) is a heterogeneous disease. Distinct molecular subtypes of UC have been identified that have clinically meaningful differences in prognosis. Several independent groups have confirmed the existence of these intrinsic subtypes with varying classification schemes and a general overall agreement of a basal-squamous and non-basal-squamous group (i.e. luminal) [1, 2]. Among the multiple classification schemes [1, 3–7], our group identified the existence of basal-like and luminal-like subtypes and derived the BASE47 (Bladder cancer Analysis of Subtype by gene Expression) RNA expression classifier to characterize the molecular subtype of patient tumors [5].

Molecular stratification of muscle-invasive urothelial cancer is of particular importance given the recent retrospective data suggesting that the basal and luminal subtypes are associated with differential response to current therapies, including platinum-based chemotherapy and immune checkpoint inhibition [8–10]. The basal subtype, in particular, may be more responsive to neoadjuvant chemotherapy but associated with worse overall prognosis compared to the luminal subtype [8]. The basal subtype is also enriched in females, and it is not known if it is enriched in black patients, such as in breast cancer [11]. Despite recent advances in treatment, suboptimal outcomes in muscle-invasive urothelial cancer remain, and patient selection for treatment, potentially incorporating molecular subtyping, is clearly needed.

The ability to efficiently and accurately determine molecular subtype in UC would enable incorporation into clinical trial design and translational research efforts to improve patient selection strategies for treatment. The original BASE47 classifier, which sorts UC tumors as basal-like or luminal-like was developed from microarray and RNAseq data, and subtyping in this manner can be cumbersome and expensive [5]. A fast, cost effective, clinically useful, fully quantitative, and precise method of molecular subtyping is therefore needed. As an example, the PAM50 subtype classifier in breast cancer has resulted in widespread clinical utility with development of a risk of recurrence model to inform clinical care and prognosis [12, 13].

The NanoString nCounter system uses fluorescent barcodes tagged to individual nucleic acid molecules (including mRNA) that are captured, imaged and counted to generate a quantitative analysis of as many as 800 different genes in a single reaction [14]. Routine clinical practice results in the collection of tumor as formalin-fixed, paraffin-embedded (FFPE) tissue, and prior studies have shown the NanoString system to result in more precise and accurate measures of RNA expression in FFPE compared to PCR-based techniques [15]. For these reasons, a transition from RNAseq-based subtype classification to a NanoString-based classifier is warranted. To evaluate our hypothesis that the NanoString system can be used for molecular subtyping of UC, we have designed a custom codeset using the original BASE47 gene set and developed a de novo NanoString BASE47 classifier to accurately and efficiently determine molecular subtype of UC tumors. We also used our novel dataset to investigate the question of whether there is enrichment of black patients in the basal subtype.

## Materials and methods

### Tissue samples

All included tumor tissue samples were derived from patients with high-grade muscle-invasive UC in three independent cohorts. The first set of UC tumor tissue samples (UNC-Training) included patients who underwent cystectomy at the University of North Carolina (UNC) Hospital between 2006 and 2014. The second set of samples (JHU-Validation) was collected at John Hopkins University Hospital from patients that underwent cystectomy between 2005 and 2014. The JHU cohort was specifically selected for inclusion for its enrichment of black patients. The third set of samples (UNC-Validation) included pre-treatment tumor samples from patients at UNC that underwent neoadjuvant chemotherapy and cystectomy between 2012 and 2017. Clinical information was annotated for all patients in each of the 3 cohorts, including patient demographics (which were self-reported and included age, sex, race, and smoking status) and tumor stage and grade. Data was abstracted from medical records from 05/2015 to 06/2015 (UNC-Training), 07/2015 to 02/2016, and 06/2016 to 10/2017 (UNC-Validation). All included patients had FFPE tissue available with adequate quality and amount, and all included patient samples were from tumors prior to any systemic therapy (ie, pretreatment samples). This study was reviewed and approved by the Institutional Review Board at UNC with an approved waiver of informed consent. All patient records were de-identified at the time of data abstraction from the electronic medical record.

### NanoString codeset design

The previously published Damrauer et al. BASE47 classifier (termed “Transcriptome BASE47 classifier” here) was considered the gold standard for molecular subtype classification in this study. Custom NanoString probes were designed to match the 47 classifier gene signature defined by Damrauer et al. [5]. A set of 4 housekeeping genes was selected from the BASE47 training dataset [5] based on their low coefficients of variance. The probeset verification was carried out using NanoString’s standard custom codesets, consumables, and assay procedures. Any probe that did not show expression above background across a test set of tumor samples was redesigned.

### RNA extraction and expression analysis

RNA was isolated from macrodissected 10uM FFPE slides from all patient samples using the Roche High Pure RNA Paraffin Kit according to manufacturer’s protocol. Isolated RNA was eluted in 50uL volume and tested using a Nanodrop One spectrophotometer. RNA samples that met our sequencing specifications for concentration (>15 ng/uL) and purity (OD 260/280 & 260/230 nm >1.6) were run on the NanoString nCounter MAX system according to specifications in the Preclinical Genomic Pathology Core at UNC-Chapel Hill and analyzed using local post-processing.

Counts of each barcode were summarized using the Nanostring nCounter system. In the negative control probes (spiked in to account for nonspecific binding), a baseline expression of 1 read was set, after which the geometric mean of the negative control probes was calculated for each sample. All housekeeping and BASE47 endogenous genes were scaled to the geometric mean of the negative control probes within the sample (the expression of all genes with values below the threshold are set to 0). Any samples that had any housekeeping gene (*AMMECR1L*, *SRPRA*, *XRCC6*, *EIF2B4*) expression below the geometric mean of the negative control probes were removed from the dataset for quality control purposes (2 samples from the UNC-Validation dataset were removed), as the barcode counts from the sample are unreliable, and subsequently resubmitted for NanoString nCounter processing. For each sample, the geometric mean of the housekeeping genes was calculated, a normalization factor (100/[housekeeping geometric mean]) was derived, and all endogenous BASE47 gene expression values were multiplied by the normalization factor to control for total signal across samples. Relative expression of classifier genes were log transformed and the resulting expression vector was input into the BASE47 PAM classifier.

For RNA sequencing, a minimum of 2 μg of total RNA was isolated from FFPE tissues. Extracted RNA was converted to double-stranded cDNA, and sequencing adapters were ligated by using the Illumina RNA Access Library Prep Protocol (Illumina). Samples were sequenced by paired-end, 150-bp sequencing on an Illumina NextSeq500. Sequence reads were aligned to the human reference genome (hg38) using MapSplice [16] and quantified with RSEM [17]. RNA sequencing data were normalized for variation in read counts, log_2_ transformed, and median centered before analysis. When combining data sets, no batch effect correction analysis was used as the samples were sequenced on the same machine with the same protocol, and we did not see obvious differences in gene expression values between datasets.

### Classifier testing and validation

Subtype classification was run using a PAM classifier with the pamr package v1.56.1 in R Studio v3.5.1 to derive both subtype calls and correlations to the basal and luminal centroid for each sample and subtyping method (original Transcriptome BASE47 classification and new NanoString BASE47 classification). The McNemar test was performed to test the concordance between the various described BASE47 classifier models. Heatmaps were generated with the ComplexHeatmap v2.6.0 package in R Studio, and all clustering was performed using average linkage clustering with a centered correlation similarity metric. Coefficients of Variance were calculated to assess repeatability. Pearson correlation coefficients and significance metrics across NanoString and RNASeq analyses were calculated where appropriate. Monte-Carlo cross-validation was performed to develop the NanoString BASE47 classification. This training utilized the UNC-Training dataset with 2/3 of the samples randomly being assigned to the training matrix and the remaining 1/3 assigned to a testing matrix, with 10,000 iterations of cross-validation to estimate expected performance. The verified BASE47 centroids for both the NanoString and RNASeq training matrices were calculated and visualized using the pamr package v1.56.1 in R Studio. For all statistical tests, a p-value ≤ 0.05 was considered statistically significant. Code associated with the publication is accessible within the Kim lab repository at https://github.com/kimlabunc/Kardos_Rose_Nanostring. Associated sequencing data has been deposited in the NCBI GEO repository and can be accessed at GEO160693.

## Results

### Technical development and validation of the BASE47 NanoString probeset

Patient samples used in this study were collected from three independent datasets: the UNC-Training (n = 52), UNC-Validation (n = 40), and JHU (n = 23), for a total of 115 samples of which n = 105 had successful high quality RNA extraction. All included samples were pre-chemotherapy treatment, high-grade UC and patient characteristics were representative of patients with UC in the general population (Table 1). The JHU dataset was enriched in black patient samples to determine whether there was enrichment of black patients within a specific subtype.

**Table 1:** Clinical characteristics by cohort for samples used for classifier training and validation.

Characteristic		UNC-Training	JHU-Validation	UNC-Validation
	n	52	23	40
Age				
	Mean	67.7	65.9	63.6
	St Dev	10.2	11.8	9.6
Grade				
	High	52 (100%)	23 (100%)	40 (100%)
Sex				
	Male	32 (62%)	16 (70%)	28 (70%)
	Female	20 (38%)	7 (30%)	12 (30%)
Race				
	White	47 (90%)	10 (43%)	33 (82%)
	Hispanic	1 (2%)	0 (0%)	1 (3%)
	Black	4 (8%)	13 (57%)	6 (15%)
Smoking Status				
	Current	15 (29%)	7 (30%)	13 (32%)
	Former	29 (56%)	11 (48%)	17 (43%)
	Never	8 (15%)	5 (22%)	10 (25%)
Prior NMIBC				
	Yes	15 (29%)	unknown	unknown
	No	37 (71%)	unknown	unknown
Prior BCG				
	Yes	11 (21%)	6 (26%)	unknown
	No	41 (79%)	17 (74%)	unknown
pTstage				
	Tis	0	0	1 (3%)
	T1	1 (2%)	0 (0%)	0 (0%)
	T2	21 (40%)	9 (40%)	12 (30%)
	T3	25 (48%)	7 (30%)	20 (50%)
	T4	5 (10%)	7 (30%)	7 (17%)
pNstage				
	N0	44 (85%)	12 (52%)	27 (68%)
	N1	2 (4%)	5 (22%)	4 (10%)
	N2	1 (2%)	6 (26%)	5 (13%)
	N3	1 (2%)	0	2 (5%)
	NX	3 (6%)	0	2 (5%)
Secondary Histology				
	None (Pure UC)	41 (79%)	15 (65%)	28 (70%)
	Squamous	1 (2%)	6 (26%)	7 (18%)
	Micropapillary	unknown	1 (4%)	3 (8%)
	Other	10 (19%)	1 (4%)	2 (5%)

Abbreviations: NMIBC, non-muscle invasive bladder cancer; BCG, Bacillus Calmette-Guerin; UC, urothelial carcinoma.

RNA was extracted from all 52 FFPE samples in the UNC-Training dataset. We first ran technical replicates (n = 2) of the same sample on the BASE47 NanoString probeset and found that RNA expression by NanoString was highly replicable across runs with a median coefficient of variance of 0.09 across genes and no gene having a coefficient of variance above 0.6 (S1A Fig). This high level of technical replicability is consistent with prior work [15]. We next ran all 52 samples from the UNC-Training set of tumors on the BASE47 NanoString probeset. We verified that the designated housekeeping genes used for normalization across samples had a lower variation in expression across samples than the BASE47 genes (S1B Fig) to confirm their validity as normalizing housekeeping genes.

### The transcriptome BASE47 classifier does not work with NanoString-derived BASE47 expression data

**Fig 1:**
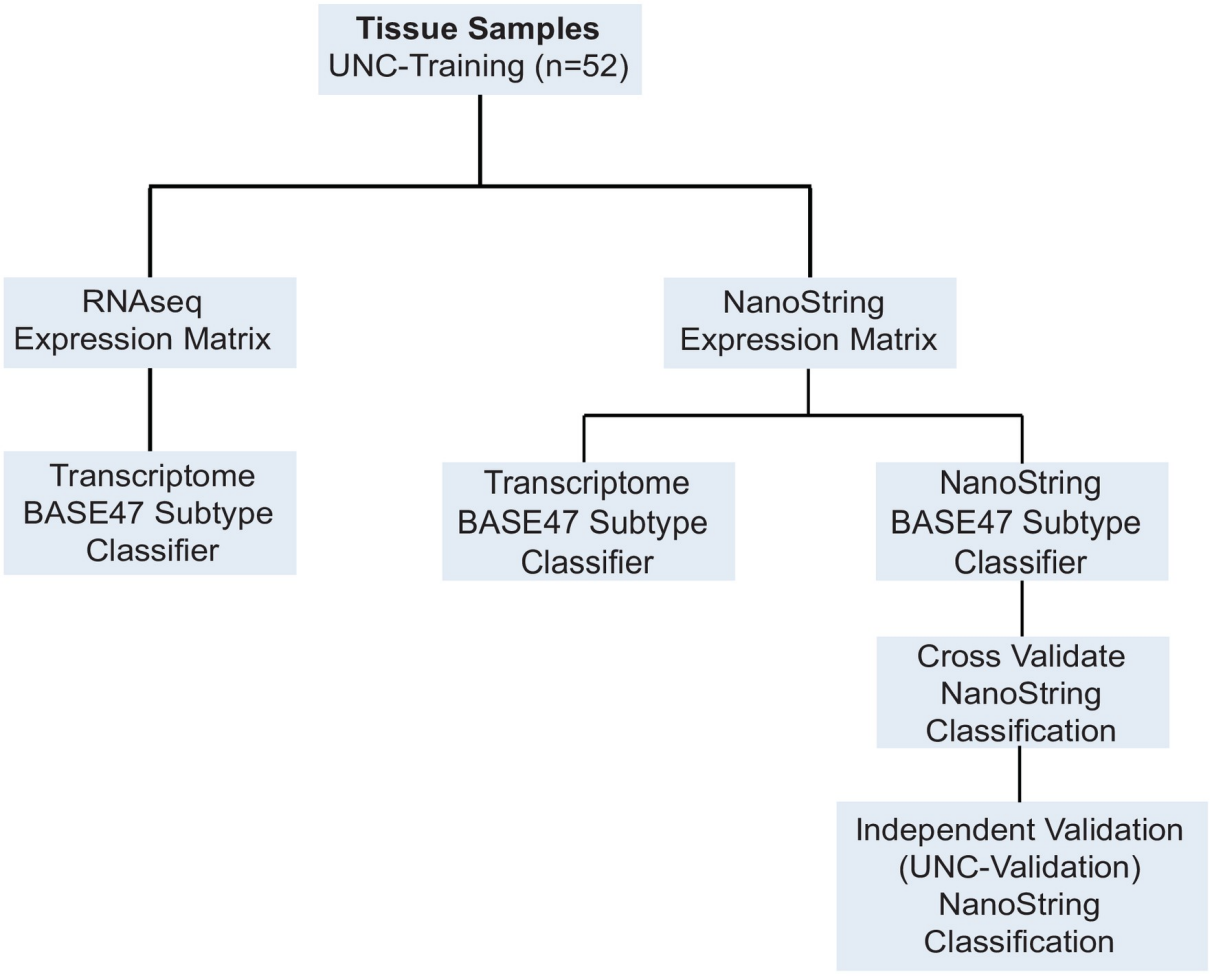
Workflow for analysis of the UNC-Training dataset. RNA from UNC-Training set samples (n = 52) were profiled by both RNAseq (RNA Access) and NanoString nCounter assay for the BASE47 probeset. The previously published Transcriptome BASE47 classifier was applied to the RNAseq data as well as the NanoString-derived BASE47 gene expression values. A NanoString BASE47 classifier was developed for NanoString nCounter expression values, tested by Monte Carlo cross validation, and independently validated on the UNC-Validation dataset.

**Fig 2:**
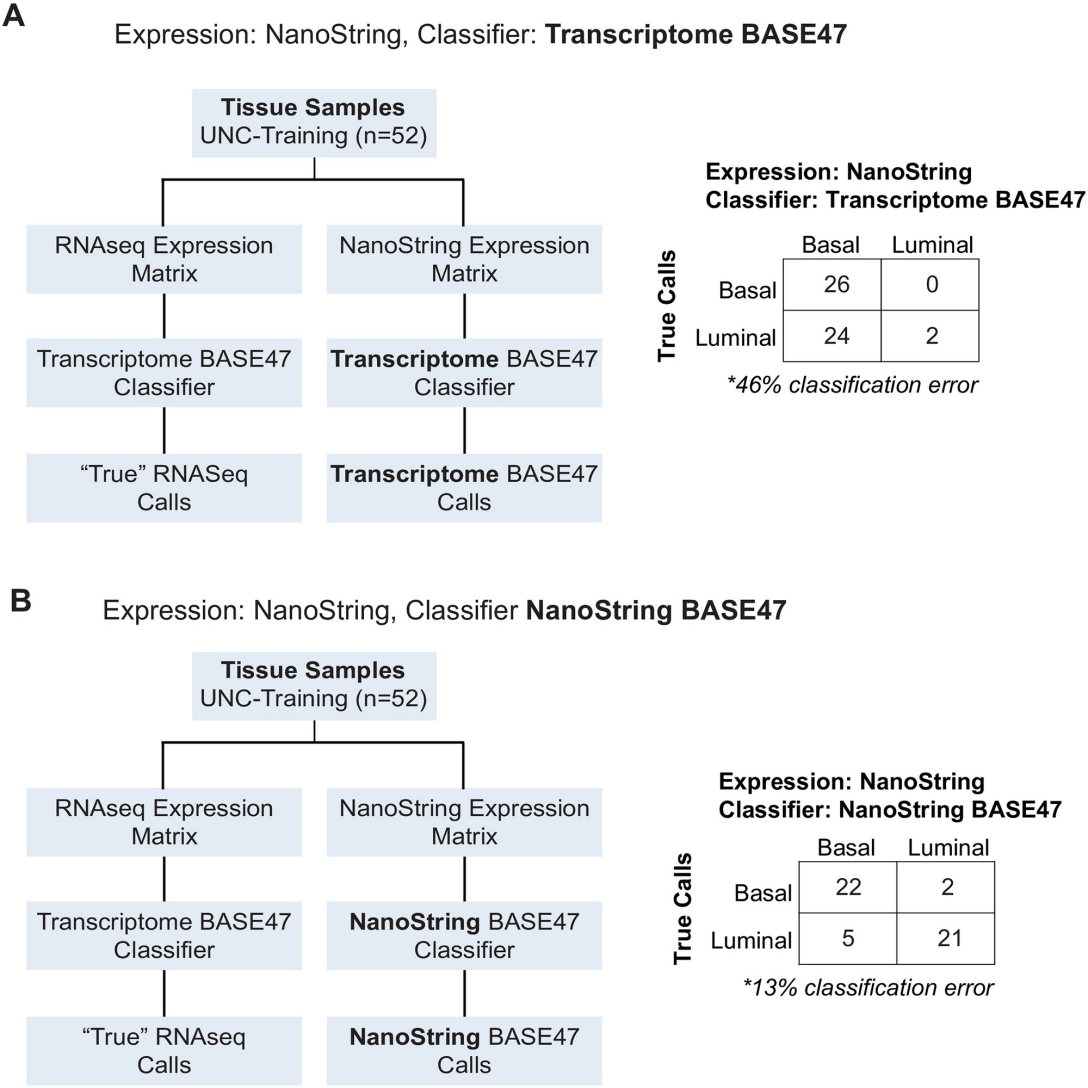
Development of a NanoString BASE47 classifier that performs on NanoString derived RNA expression values. RNA from UNC-Training set samples (n = 52) were profiled by both RNAseq (RNA Access) and NanoString nCounter assay for the BASE47 probeset to generate RNAseq and NanoString expression matrices respectively. “True” biologic calls were assigned by applying the previously published Transcriptome BASE47 classifier to the RNAseq data. **(A)** Application of the Transcriptome BASE47 classifier to the NanoString expression data resulted in a 46% misclassification error. **(B)** Application of the newly derived NanoString BASE47 classifier to NanoString expression data resulted in only 13% misclassification error (relative to the “True” biologic subtype designations).

The RNA from the UNC-Training samples was run simultaneously on both the NanoString nCounter platform (with our BASE47 probeset) and sequenced by RNASeq (Fig 1). Subtype calls were made using our original Transcriptome BASE47 classifier on the processed RNAseq data. These calls were considered the biologically ‘true’ subtype of each sample [5]. We first classified the 52 samples in the UNC-Training dataset using their NanoString derived BASE47 RNA expression values with the original Transcriptome BASE47 classifier [5]. This was a highly inaccurate method of classification as 24 of 52 (46%) samples were incorrectly classified (Fig 2A). This method of classification dramatically overcalled basal tumors, classifying all but 2 samples as basal.

### Development of a NanoString BASE47 classifier

**Fig 3:**
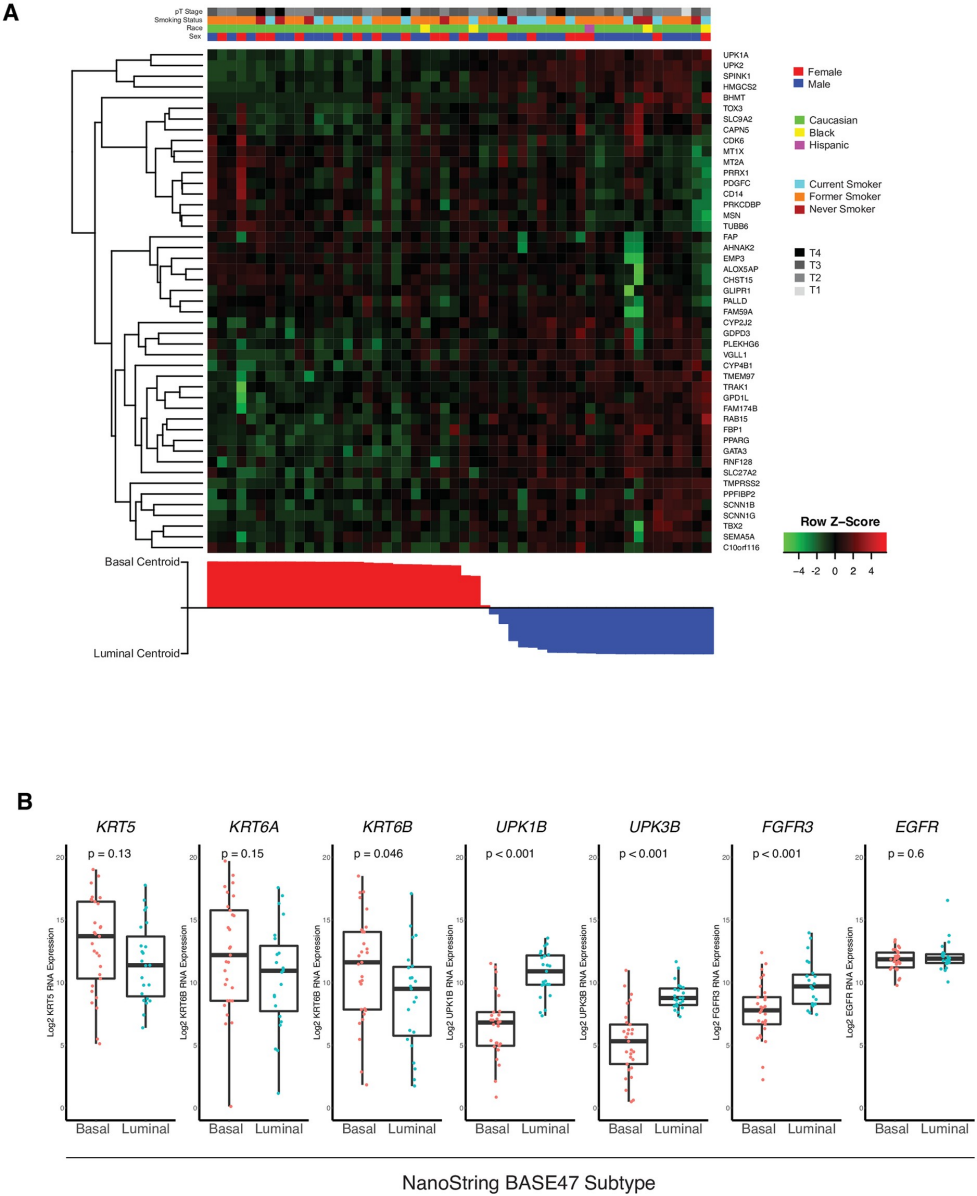
Heatmap of BASE47 genes on samples supervised by correlation to basal centroid. **(A)** Samples were supervised by correlation to the basal centroid (NanoString BASE47 classifier applied to NanoString expression) and clustered on BASE47 gene expression values derived by NanoString nCounter assay. Annotation of sex, race, smoking status, and T stage are indicated. **(B)** Log2 normalized RNA expression of canonical basal and luminal genes based on NanoString subtype demonstrate expected basal and luminal expression patterns.

Because of the inaccuracy of applying NanoString derived BASE47 expression values to our Transcriptome BASE47 classifier, we set out to retrain a NanoString BASE47 classifier to accurately make subtype calls on NanoString derived RNA expression values. Indeed, scientific best practices dictate that a de novo retraining of the BASE47 bladder cancer intrinsic subtype classifier should be performed on NanoString derived expression values in order to develop the most accurate and robust classifier. To this end, we used the “true” subtype calls from the RNAseq and Transcriptome BASE47 classifier as the supervising variable to identify new luminal and basal centroids from the corresponding NanoString derived RNA expression. Application of the “NanoString BASE47 classifier” to the UNC-Training dataset using NanoString derived RNA expression values, as expected, dramatically improved accuracy relative to the use of the Transcriptome BASE47 classifier as only 7 of the 52 samples (13%) were incorrectly classified (Fig 2B). The 13% training error of the NanoString BASE47 classifier was significantly better than the 46% training error when the UNC-Training set was classified using NanoString expression values with the Transcriptome BASE47 classifier (p<0.001, McNemar test). The NanoString BASE47 classifier not only provides a basal and luminal subtype call, but a scaled distance from 0 (luminal) to 1 (basal) to each centroid. There was not a significant correlation between the centroid values of the Transcriptome BASE47 classifier from RNAseq data and the centroid values of the Transcriptome BASE47 subtype classifier from NanoString derived RNA expression data (S2A Fig). In contrast, there was a strong correlation between centroid values of the Transcriptome BASE47 classifier calls using RNAseq data and the centroid values of NanoString BASE47 subtype classifier calls using NanoString derived RNA expression data (S2B Fig, R = 0.88, p<0.001). Examination of clinical characteristics (T stage, sex, ethnicity, and smoking status) found no association of clinical variables with subtype classification (Fig 3A and S3 Fig).

We were concerned that predicting subtype calls onto the same dataset that was used to derive the NanoString BASE47 classifier (UNC-Training) could bias our training error through self-validation. To rule out this potential bias, we ran a Monte Carlo cross-validation by randomly splitting the UNC-Training dataset into a training matrix (2/3 patients) and used the resulting centroids to predict onto the remaining 1/3 of the samples (S4A Fig). The Monte Carlo sampling was run 10,000 times and compared to the ‘true’ subtype calls of each sample. The Monte Carlo simulation resulted in a 16.2% training error that was not significantly different from the 13% originally derived training error (X^2^ p = 0.96), indicating that our dataset was not biasing the accuracy of the subtype classification (S4B Fig).

To further verify the accuracy of our basal and luminal subtype calls we examined the NanoString derived RNA expression of the BASE47 genes in the UNC-Training dataset. A heatmap of the 52 tumors from the UNC-Training dataset supervised by distance to the basal centroid in the Nanostring BASE47 classifier demonstrated that there were very distinct basal and luminal nodes of BASE47 genes, consistent with previous analyses of genes in the Transcriptome BASE47 classifier (Fig 3A) [5]. In addition, examination of canonical basal (KRT5, KRT6A) and luminal (UPK1B, UPK3B, FGFR3)-like genes by NanoString subtype demonstrated patterns consistent with basal-like and luminal-like differentiation (Fig 3B).

### Validation of NanoString BASE47 classifier on UNC validation dataset

**Fig 4:**
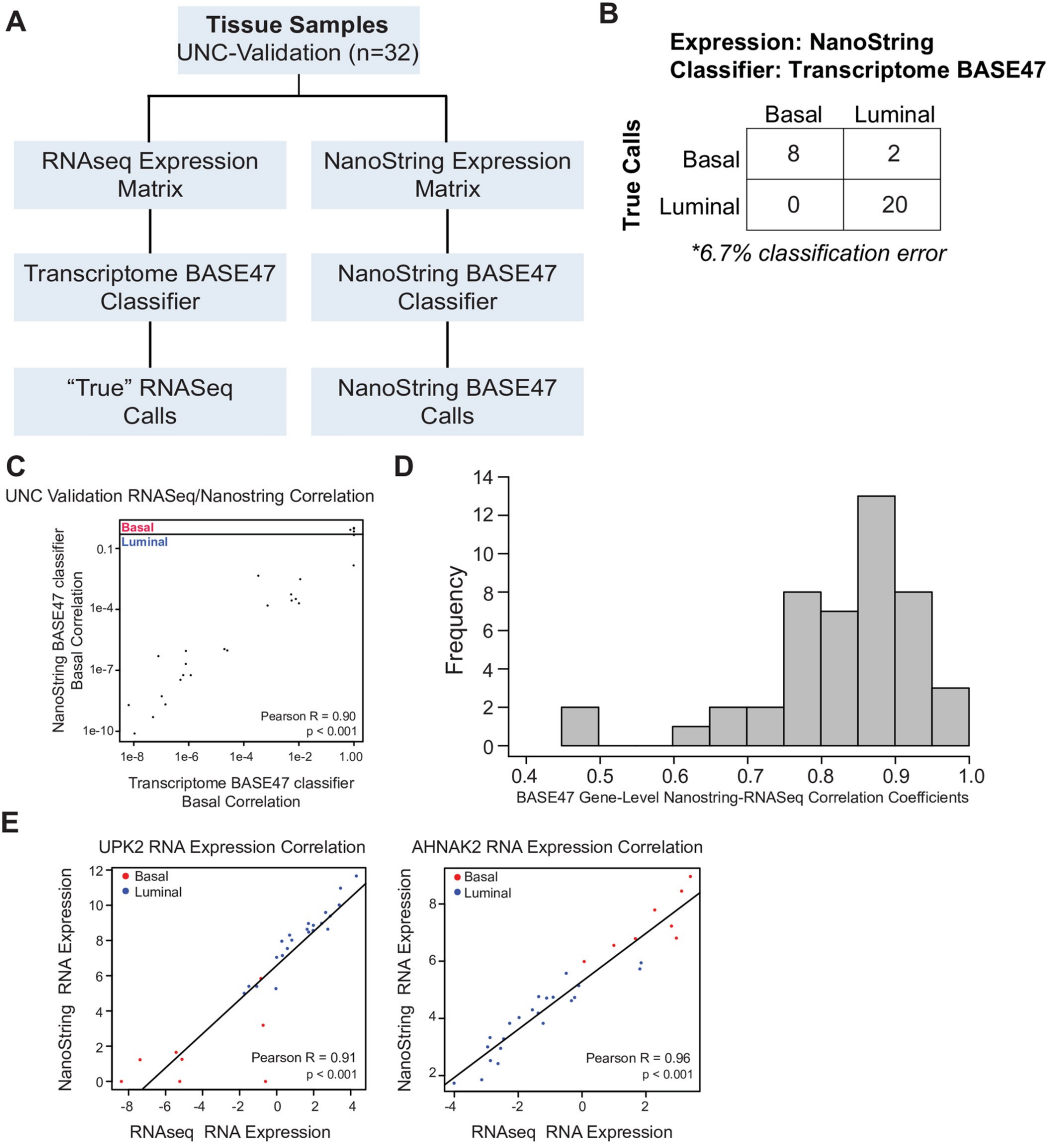
Validation of NanoString BASE47 classifier on UNC-Validation dataset. **(A)** UNC-Validation samples were profiled by both RNAseq (RNA Access) and NanoString nCounter assay for the BASE47 probeset to generate RNAseq and NanoString expression matrices respectively. “True” biologic calls were assigned by applying the previously published Transcriptome BASE47 classifier to the RNAseq data. NanoString BASE47 calls were determined by application of the newly derived NanoString BASE47 classifier to NanoString expression data. **(B)** Application of the newly derived NanoString BASE47 classifier to NanoString expression data resulted in only 6.7% misclassification error (relative to the “True” biologic subtype designations). **(C)** The basal centroid correlation values derived from application of the Transcriptome BASE47 classifier to RNAseq data (X axis) and the NanoString BASE47 classifier applied to NanoString expression data (Y axis) were significantly correlated, R = 0.90, p < 0.001. **(D)** Histogram of frequency of correlation coefficients of BASE47 genes between basal centroid correlation values derived from application of the Transcriptome BASE47 classifier to RNAseq data and the NanoString BASE47 classifier applied to NanoString expression data. **(E)** Scatter plots of expression values of a representative luminal (*UPK2*) and basal (*AHNAK2*) BASE47 genes derived from NanoString and RNAseq.

In order to further validate our new NanoString BASE47 classifier, we examined the accuracy of the NanoString BASE47 classifier on a set of independent tumors (n = 40, UNC-Validation dataset). We performed RNAseq on 30 of the 40 samples in the UNC-Validation dataset for which we were able to extract sufficient RNA and again ran RNAseq and NanoString in parallel to determine Transcriptome BASE47 and NanoString BASE47 classifier subtype calls respectively (Fig 4A). Only 2 of the 30 samples were incorrectly classified for a 6.7% classification error (Fig 4B). This demonstrates independent validation of the NanoString BASE47 classifier. Furthermore, the basal centroid correlation values derived from NanoString BASE47 classifier using NanoString derived expression values were significantly correlated with the basal centroid values obtained from the Transcriptome BASE47 classifier using RNAseq expression (Fig 4C, R = 0.90, p<0.001). Further supporting the reliability of the NanoString nCounter method as a measure of gene expression, all individual genes within the BASE47 gene list had a significant correlation between the gene expression as measured by RNAseq and NanoString platforms (Fig 4D). For example, *UPK2* (luminal gene) and *AHNAK2* (basal gene) are shown (Fig 4E, p≤0.001). These data in aggregate demonstrate that our NanoString BASE47 classifier and centroid correlations are representative of the previous subtype classification methodology (Transcriptome BASE47 classifier).

We examined whether there were differences in overall survival (OS) in basal and luminal tumors in the combined cohort of patients based on their NanoString BASE47 subtype and did not see any difference in OS by molecular subtype (S5 Fig). Prior work has shown that patients with basal-like tumors who are not treated with cisplatin-based neoadjuvant chemotherapy have shortened overall survival [4, 8]. In contrast, it appears that patients with basal-like tumors treated with neoadjuvant chemotherapy have equivalent overall survival [8]. In our cohort of patients, approximately 60% received neoadjuvant chemotherapy. This may account for the relatively equivalent OS in patients with basal and luminal tumors.

### Black patients with UC are not enriched in the basal subtype

**Fig 5:**
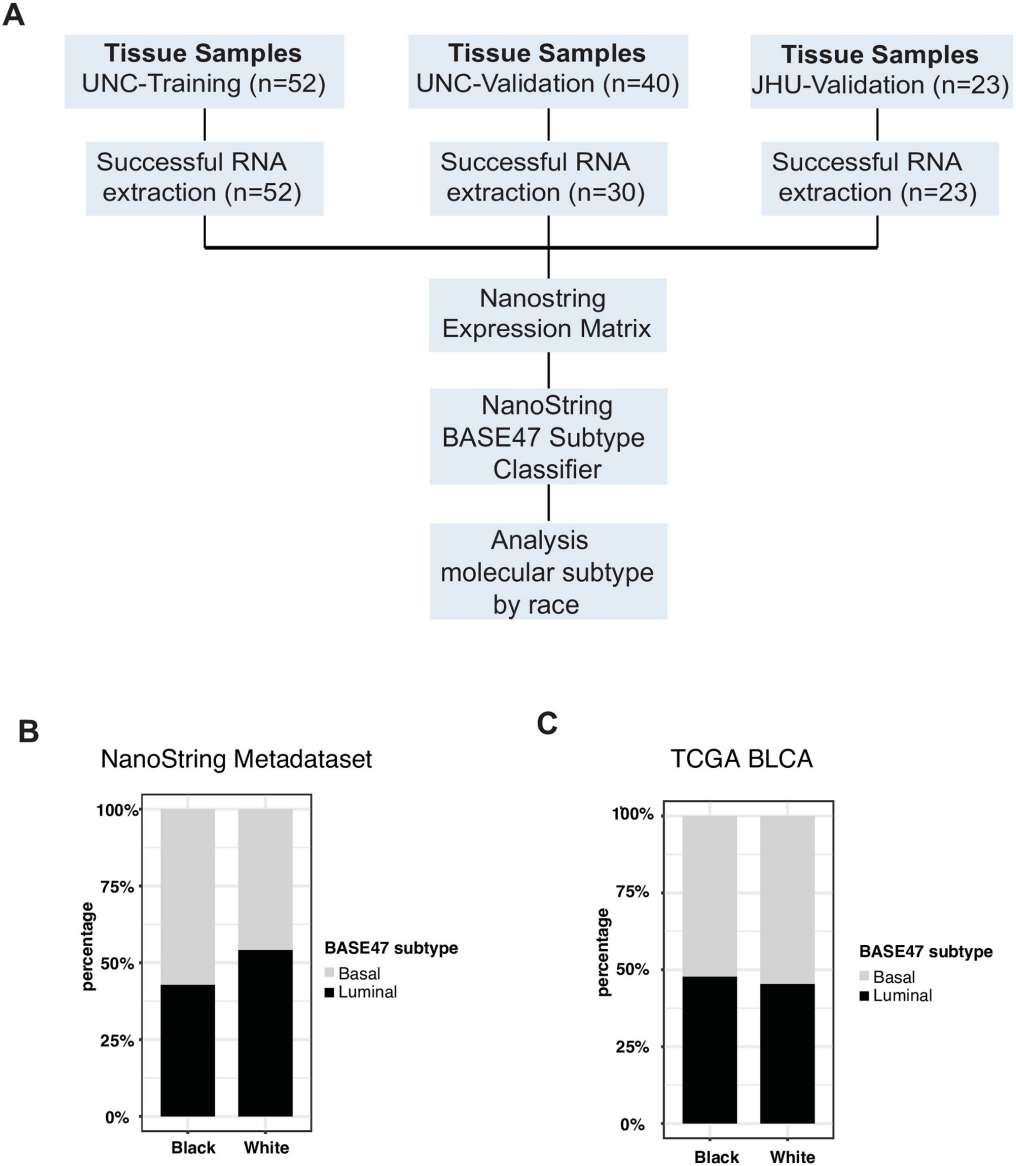
Black patients are not enriched in basal-like bladder cancer. **(A)** Workflow of analysis of the samples used to examine the molecular subtype designations of bladder cancers in black patients. **(B)** Percentage of patients with each subtype by ethnicity in the UNC/JHU metadataset. **(C)** Percentage of patients with each subtype by ethnicity in the TCGA BLCA.

Prior work in breast cancer has shown that black women are enriched in the basal subtype of breast cancer [11]. Given the parallels between the molecular subtypes of breast and bladder cancer [4, 5], we hypothesized that black patients might also be enriched in the basal subtype of urothelial bladder cancer. We obtained a cohort of high-grade, muscle-invasive bladder cancer patients from Johns Hopkins (JHU) that were enriched for black patients (Table 1). RNA was extracted from all 23 samples and successfully run on the BASE47 NanoString nCounter assay (Fig 5A).

We examined NanoString BASE47 subtype calls from the compilation of our UNC-Training (n = 52), UNC-Validation (n = 30), and JHU (n = 23) datasets (UNC/JHU metadataset) and found no significant enrichment of black patients in the basal-like subtype (13 of 21 [62%] black patients were classified as basal-like [Fisher’s exact p = 0.24]) [Fig 5B]. An examination of the TCGA BLCA dataset also found no significant enrichment of black patients in the basal subtype (12 of 23 [52%], Chi-square p = 0.08). Therefore, at least in the cohorts examined here, black patients with bladder cancer do not appear to have an increased prevalence of basal-like bladder cancer.

## Discussion

Here we have derived and validated a NanoString BASE47 classifier that accurately classifies high-grade, urothelial bladder cancers using NanoString derived RNA expression values and BASE47 centroid values distinct from our previously published Transcriptome BASE47 classifier [5]. We also find no enrichment of black patients in the basal-like subtype in either the TCGA BLCA dataset or our combined cohort.

The original BASE47 classifier was derived on microarray and RNAseq platforms. While these platforms provide full transcriptomic expression data, the delayed time it takes to process samples and the increased cost associated with sample processing make the combination of RNAseq and Transcriptome BASE47 classifier cumbersome for clinical implementation. The NanoString nCounter assay and the NanoString BASE47 classifier provide a faster turnaround time, a lower cost per sample, and maintain the accuracy of the original subtype classifier. The time to library prep (particularly from FFPE derived RNA) and sequence a sample on the original BASE47 classifier takes upwards of a week, while a sample can be processed and classified on the NanoString platform in as quickly as 24–48 hours. Furthermore, sequencing a sample via RNAseq costs approximately 5 times the cost of the NanoString nCounter assay. Transitioning to a NanoString platform therefore provides a faster and more cost-effective pathway to subtype classification that can be incorporated into translational research efforts and in the future, potential treatment decision-making.

Another key advantage of our new NanoString BASE47 classifier is the ability to classify single samples into luminal-like and basal-like categories. The original Transcriptome BASE47 classifier was developed on a median-centered dataset and was originally designed to be used to classify cohorts of patient sample data and as a result is not suitable for the classification of individual patient samples. The Transcriptome BASE47 classifier requires each researcher to select an appropriate, representative population and devise a method for gene centering in order to normalize the population to the original published training population to minimize bias and inaccuracy in subtype classification. In contrast, our NanoString BASE47 classifier uses fixed expression values held stable across samples relative to the trained centroids with no inter-sample processing or normalization. Therefore, no additional cohort or platform normalization is required, providing a stable subtyping algorithm that can be applied to a single patient regardless of biases in clinical characteristics, allowing for more widespread clinical and translational use of the NanoString BASE47 classifier in decentralized laboratories. Recently, given the multiple classification schemas for molecular subtypes in UC, efforts have been made to define a consensus classifier. Our analysis is an important step forward to clinical use of a Nanostring-based classifier, and opens the opportunity for further validation with other classification schemes. In particular, a recently published consensus paper outlined six molecular subtypes based on RNA transcriptome data, and a logical future extension of our current work would be validation of a NanoString classifier for the consensus subtypes for easy and wide clinical implementation.

It is pertinent to put our NanoString classifier in the context of other techniques and tools for classification of molecular subtypes in UC. There are several other transcriptome based classifiers including a consensus classifier that identified six subtypes and a method for single sample classification [2]. Our analysis only validates the use of a NanoString classifier for the two subtype transcriptome BASE47 classifier, but demonstrates a proof of principle that can later be expanded to other classification schemes. There has also been recent interest in immunohistochemical (IHC) subtype classification, which can provide an affordable, fast, and clinically accessible results with similar advantages to the NanoString platform. Indeed, Guo et al. demonstrated that IHC classification using GATA3 and KRT5/6 is a viable method of classification for most tumors [18], although the accuracy was less than is described in our study with NanoString.

We were surprised to not detect enrichment of black patients in the basal-like subtype of bladder cancer given the similarities between breast and bladder cancer molecular subtypes and the well documented enrichment of black women with breast cancer in the basal-like subtype [11]. Our inability to detect enrichment of black patients in the basal-like subtype of bladder cancer could represent a lack of statistical power to detect such a difference, as this dataset also did not detect an enrichment in the basal subtype in females, which has been shown previously [1]. However, we do feel that our sample size is sufficient to detect a clinically meaningful difference in subtype distribution among races. For comparison, the Carolina Breast Cancer Study examined 496 women with breast cancer, of which 196 were black and found an increased prevalence of basal-like breast cancer in black women [odds ratio (OR) of 2.1 (p = 0.004)] [11]. Alternatively, the lack of enrichment of black patients in the basal-like bladder cancer subtype could certainly reflect a true difference in the molecular epidemiology between breast and bladder cancer or a yet to be defined interaction between race, sex, and molecular subtype (since our black patients included both males and females).

The enclosed analysis demonstrates feasibility and validation of a NanoString based classifier in urothelial cancer but is not without limitations. While our sample size is adequate to demonstrate accuracy of our classifier, our analysis is not sufficiently powered to demonstrate association with survival, as has been done with prior classifiers in urothelial cancer. Additionally, we were unable to demonstrate that the molecular subtypes have different response to chemotherapy and differential survival given the heterogeneity of our patient population. However, given the low classification error in our training and validation datasets, we feel this classifier advances molecular subtype classification toward clinical utility in UC treatment.

## Conclusions

The development of a fast, cost-effective, and accurate platform for BASE47 subtype classification will now enable further translational research efforts and move the field forward toward the incorporation of the basal and luminal molecular subtype classification into clinical use. It also opens up the possibility of transitioning other urothelial cancer subtyping schemas for further analysis to better inform the clinical care of the patients.

## Supporting information

S1 Fig Technical validation of the BASE47 NanoString probeset. **(A)** Coefficient of Variance of NanoString expression across technical replicates (n = 2) on a representative bladder cancer sample. **(B)** Coefficient of Variance of NanoString expression across the UNC-Training dataset (n = 52).
(PDF)

S2 Fig Cross validation of a NanoString BASE47 PAM predictor. **(A)** Scatter plot of the correlation to the basal centroid of Transcriptome BASE47 classifier applied to RNAseq expression (X axis) versus correlation to the basal centroid of the Transcriptome BASE47 classifier applied to NanoString BASE47 expression (Y axis) demonstrating poor correlation (R = 0.25). **(B)** Scatter plot of the correlation to the basal centroid of Transcriptome BASE47 classifier applied to RNAseq expression (X axis) versus correlation to the basal centroid of the NanoString BASE47 classifier applied to NanoString BASE47 expression (Y axis) demonstrating excellent correlation (R = 0.88).
(PDF)

S3 Fig Association of clinical factors and molecular subtype. Tabulation of clinical characteristics of patients in the UNC/JHU metadataset and molecular subtype as determined by the NanoString BASE47 Subtype classifier demonstrates no association by Chi-squared testing of subtype with **(A)** T stage, p = 0.45 **(B)** race, p = 0.52 **(C)** sex, p = 0.46 and **(D)** smoking status, p = 0.37.
(PDF)

S4 Fig Schematic of Monte Carlo cross-validation of the NanoString BASE47 classifier. **(A)** The UNC-Training dataset was randomly split into a training matrix (2/3 patients) and the resulting centroids were used to predict onto the remaining 1/3 of samples. The sampling was run 10,000 times. **(B)** The confusion matrix denotes the percentage of the simulations that matched the ‘true’ subtype calls of each sample. There was a 16.2% classification error.
(PDF)

S5 Fig Survival analysis stratified by subtype. Kaplan-Meier estimate of overall survival (OS) stratified by molecular subtype based on NanoString BASE47 Subtype Classification demonstrated no difference in overall survival in patients with basal and luminal tumors (p = 0.61).
(PDF)
