# Supplementary figures and images for: Development and validation of a NanoString BASE47 bladder cancer gene classifier
Source: PLoS One. 2020 Dec 17;15(12):e0243935. doi: 10.1371/journal.pone.0243935 (PMC7745986; doi:10.1371/journal.pone.0243935)

S1 Fig

A

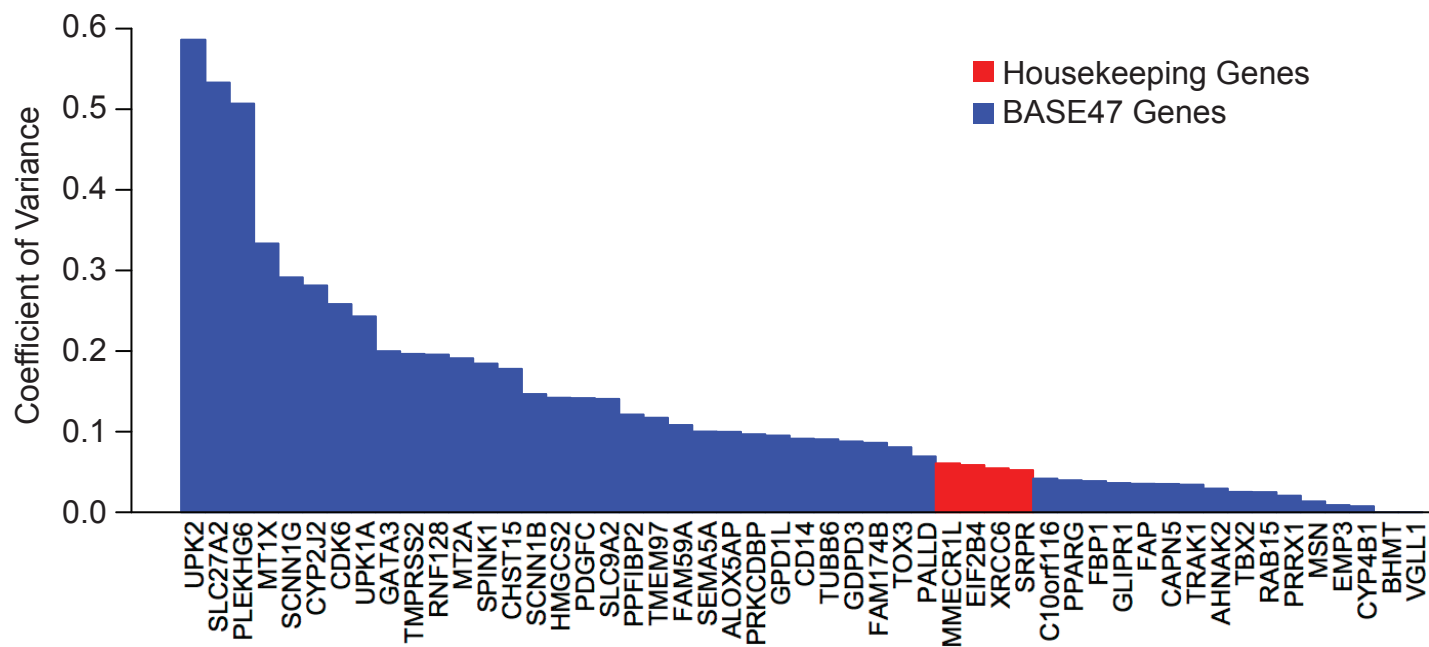

B

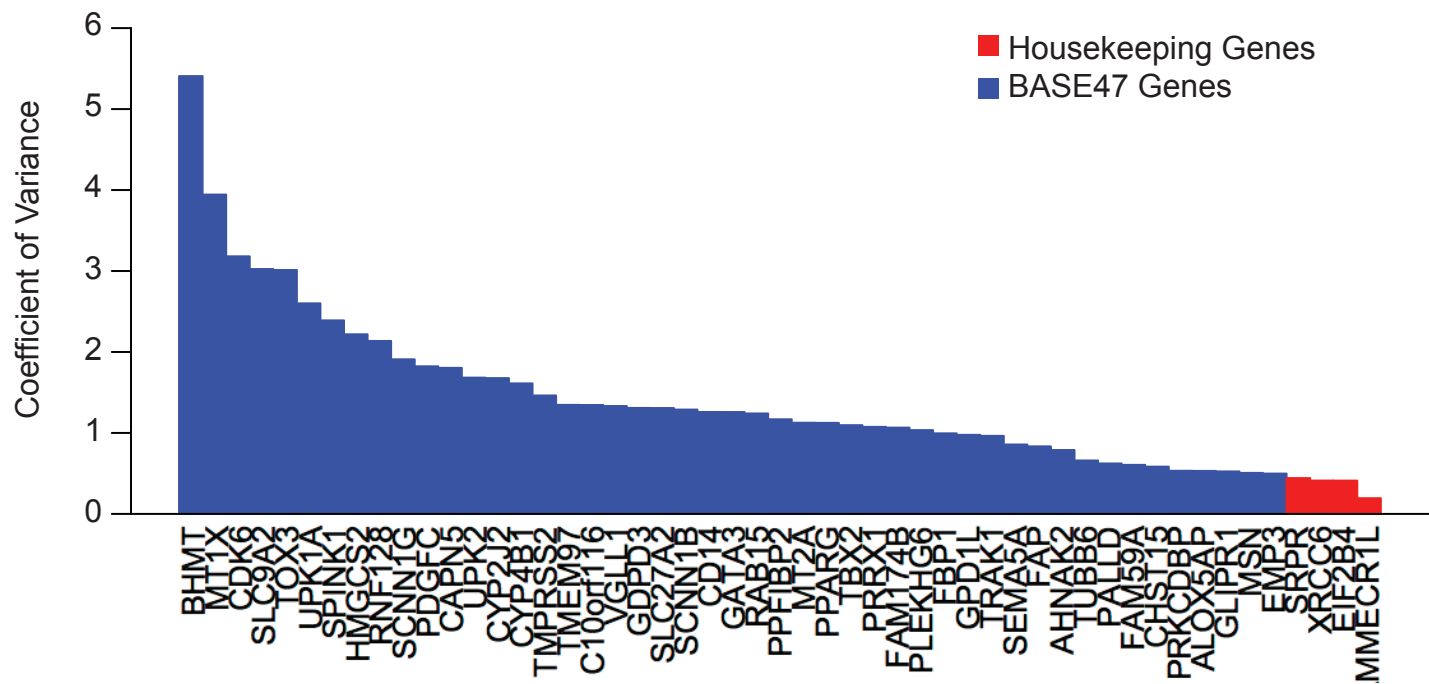

Supplement: S1 Fig — (A) Coefficient of Variance of NanoString expression across technical replicates (n = 2) on a representative bladder cancer sample. (B) Coefficient of Variance of NanoString expression across the UNC-Training dataset (n = 52). (PDF) [file pone.0243935.s001.pdf]

## S2 Fig

**A**

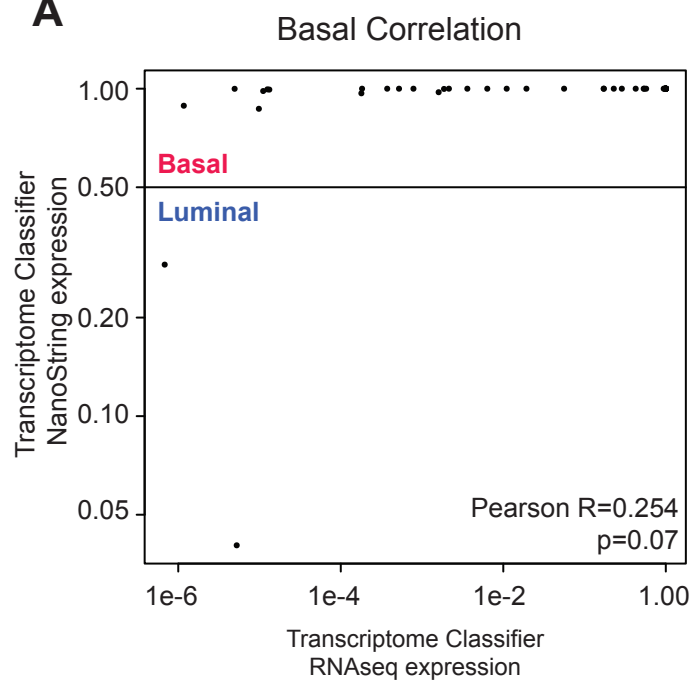

**B**

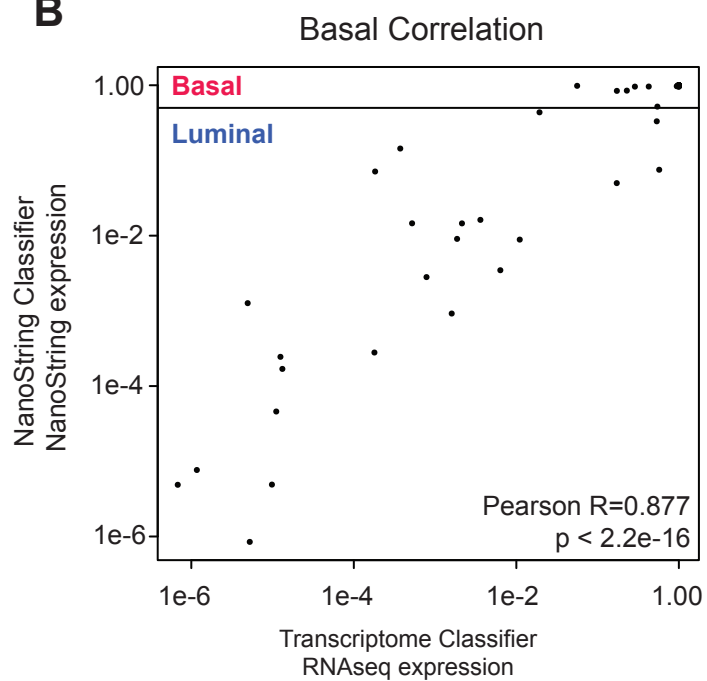

Supplement: S2 Fig — (A) Scatter plot of the correlation to the basal centroid of Transcriptome BASE47 classifier applied to RNAseq expression (X axis) versus correlation to the basal centroid of the Transcriptome BASE47 classifier applied to NanoString BASE47 expression (Y axis) demonstrating poor correlation (R = 0.25). (B) Scatter plot of the correlation to the basal centroid of Transcriptome BASE47 classifier applied to RNAseq expression (X axis) versus correlation to the basal centroid of the NanoString BASE47 classifier applied to NanoString BASE47 expression (Y axis) demonstrating excellent correlation (R = 0.88). (PDF) [file pone.0243935.s002.pdf]

S4 Fig

A

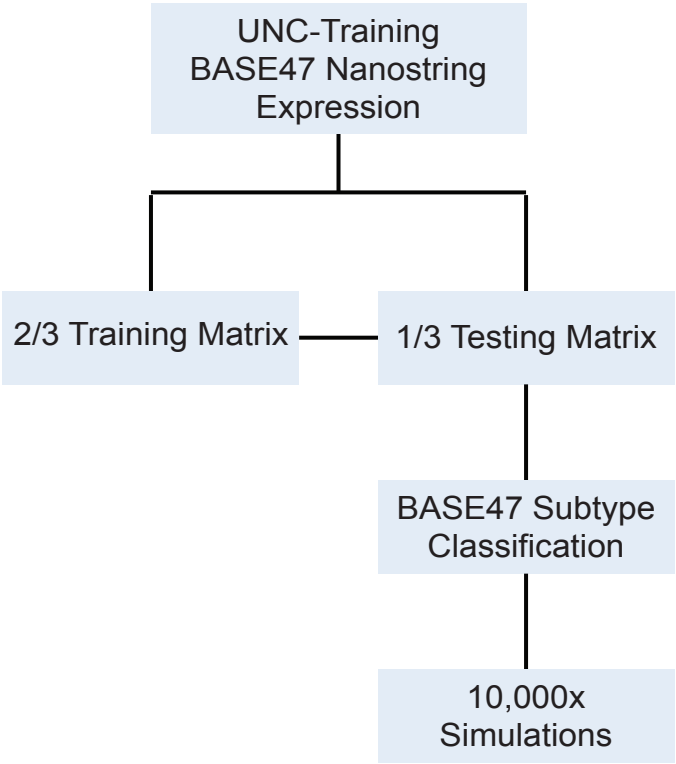

B

**NanoString Simulation**

|         | Basal | Luminal |
|---------|-------|---------|
| Basal   | 44.8% | 5.2%    |
| Luminal | 11.0% | 39.0%   |

*\*16.2% classification error*

Supplement: S4 Fig — (A) The UNC-Training dataset was randomly split into a training matrix (2/3 patients) and the resulting centroids were used to predict onto the remaining 1/3 of samples. The sampling was run 10,000 times. (B) The confusion matrix denotes the percentage of the simulations that matched the ‘true’ subtype calls of each sample. There was a 16.2% classification error. (PDF) [file pone.0243935.s004.pdf]

S5 Fig

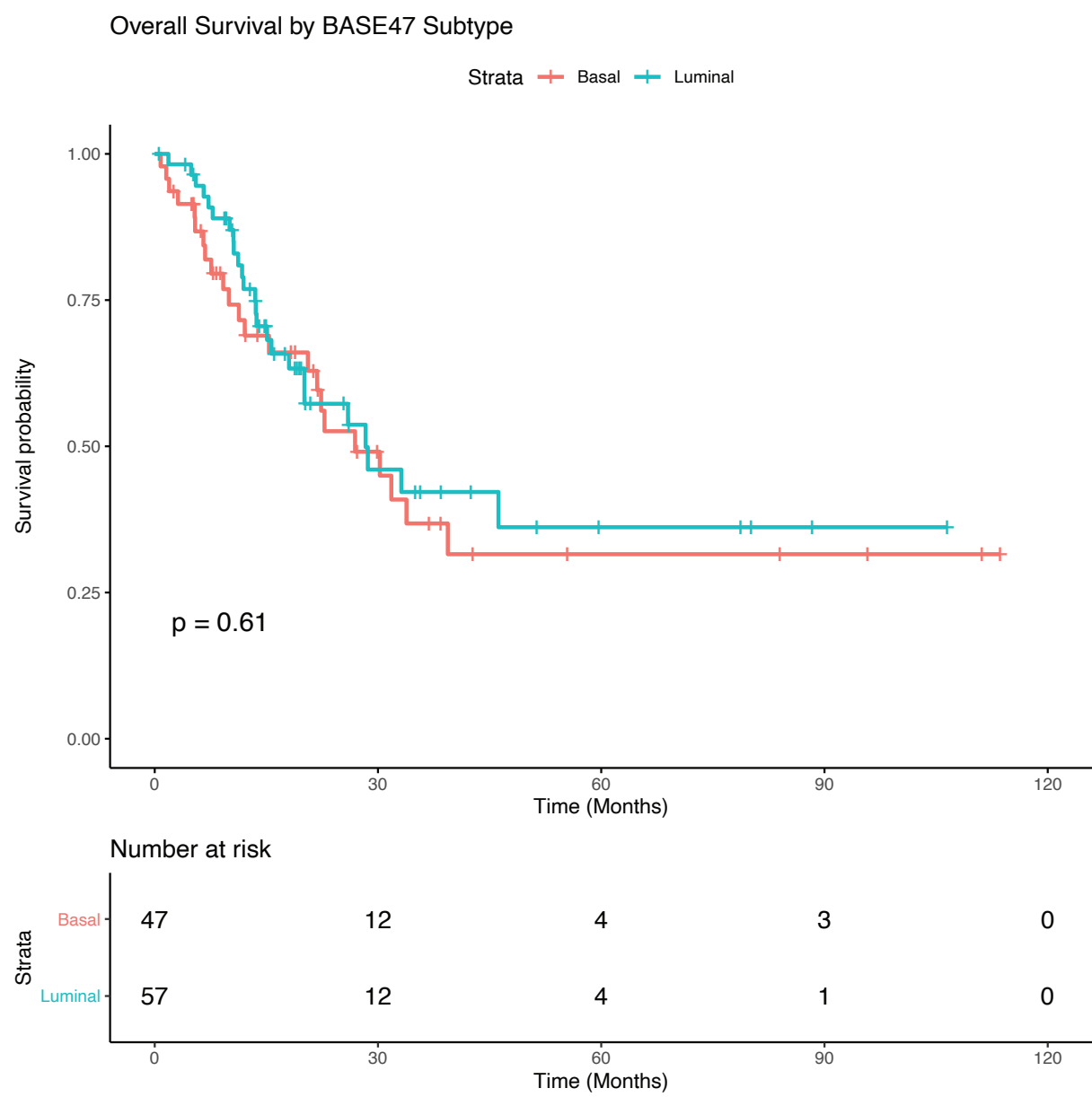

Supplement: S5 Fig — Kaplan-Meier estimate of overall survival (OS) stratified by molecular subtype based on NanoString BASE47 Subtype Classification demonstrated no difference in overall survival in patients with basal and luminal tumors (p = 0.61). (PDF) [file pone.0243935.s005.pdf]
