# Supplementary material for: Development and validation of a NanoString BASE47 bladder cancer gene classifier
Source: PLoS One. 2020 Dec 17;15(12):e0243935. doi: 10.1371/journal.pone.0243935 (PMC7745986; doi:10.1371/journal.pone.0243935)
Supplement: S3 Fig — Tabulation of clinical characteristics of patients in the UNC/JHU metadataset and molecular subtype as determined by the NanoString BASE47 Subtype classifier demonstrates no association by Chi-squared testing of subtype with (A) T stage, p = 0.45 (B) race, p = 0.52 (C) sex, p = 0.46 and (D) smoking status, p = 0.37. (PDF) [file pone.0243935.s003.pdf]

S3 Fig

A

	Basal	Luminal
**Tis**	0	1
**T1**	0	1
**T2**	14	23
**T3**	25	22
**T4**	8	10

*Chi-squared p = 0.45

B

	Basal	Luminal
**African American**	11	10
**White**	36	46

*Chi-squared p = 0.52

C

	Basal	Luminal
**Female**	19	18
**Male**	28	39

*Chi-squared p = 0.46

D

	Basal	Luminal
**Current Smoker**	11	19
**Former Smoker**	27	25
**Never Smoked**	9	13

*Chi-squared p = 0.37
